# P74 Improving penicillin allergy risk-stratification through pharmacy-led education in a specialist elective orthopaedic hospital

**DOI:** 10.1093/jacamr/dlaf230.081

**Published:** 2025-12-04

**Authors:** Hannah Coldicott, Nickisha Patel

**Affiliations:** The Royal Orthopaedic Hospital NHS Foundation Trust; The Royal Orthopaedic Hospital NHS Foundation Trust

## Abstract

**Background:**

Penicillin allergy labels are recorded in approximately 6% of the general population; however, studies indicate that up to 95% of these labels are inaccurate when patients undergo formal allergy testing. Incorrect penicillin allergy documentation leads to the use of alternative broad-spectrum antibiotics, which can contribute to antimicrobial resistance, increased healthcare costs and poorer patient outcomes. A thorough penicillin allergy history is essential to accurately risk-stratify patients as either high or low risk for true allergy, enabling the safe challenge and removal of incorrect allergy labels. The British Society for Allergy and Clinical Immunology (BSACI) provide guidelines to support this risk stratification. At the Royal Orthopaedic Hospital, a recent review of penicillin allergy documentation revealed that fewer than 3% of histories contained sufficient clinical detail to support risk stratification according to BSACI criteria, highlighting the need for targeted education.

**Objectives:**

The primary aim of this quality improvement project was to evaluate the impact of targeted education and training sessions delivered to pharmacy professionals on the quality and detail of penicillin allergy documentation. The secondary aim was to assess whether this improved documentation enhanced the ability to risk-stratify patients and identify those suitable for de-labelling.

**Methods:**

Education sessions were developed and delivered by the antimicrobial stewardship team to pharmacy staff, emphasizing the importance of capturing comprehensive penicillin allergy histories and applying BSACI risk-stratification criteria. Training included practical guidance on documenting reaction type, timing, severity and tolerance of subsequent antibiotics. Following this intervention, a retrospective review was conducted on 323 inpatient records from three orthopaedic wards over a 4 week period (May to June 2025). Patients labelled as penicillin allergic were identified (*n*=30), and their allergy documentation was reviewed against BSACI criteria to determine whether it contained sufficient information for risk stratification. The results were compared to baseline data collected prior to the educational intervention.

**Results:**

Following education and training, 47% (*n*=14) of patients with penicillin allergy labels had sufficient documentation to allow risk stratification, representing a substantial improvement from fewer than 3% before the intervention. Of these patients, 23% (*n*=7) were classified as low risk and potentially eligible for de-labelling based on their detailed allergy histories. Enhanced documentation included more comprehensive recording of allergic reactions, timelines and prior antibiotic exposures, which are critical elements for applying BSACI guidelines accurately.

**Conclusions:**

This quality improvement initiative demonstrates that targeted pharmacy-led education significantly improves the completeness of penicillin allergy documentation and increases the ability to risk-stratify patients in an elective orthopaedic hospital setting. Improved documentation supports safer, more effective antimicrobial stewardship by enabling the identification and potential de-labelling of patients with incorrect allergy labels, thereby optimizing antibiotic prescribing practices and reducing reliance on broader-spectrum agents. Future work will focus on extending this intervention to the Pre-Operative Assessment Clinic to ensure prescribers have immediate access to comprehensive allergy information at admission, facilitating timely, informed antimicrobial decision-making and improved patient outcomes.

